# Supplementary material for: The Role of Vitamin D Supplementation on Airway Remodeling in Asthma: A Systematic Review
Source: Nutrients. 2023 May 26;15(11):2477. doi: 10.3390/nu15112477 (PMC10255490; doi:10.3390/nu15112477)
Supplement: Supplementary file 1 [file nutrients-15-02477-s001.zip › nutrients-2394072-supplementary figure.pdf]

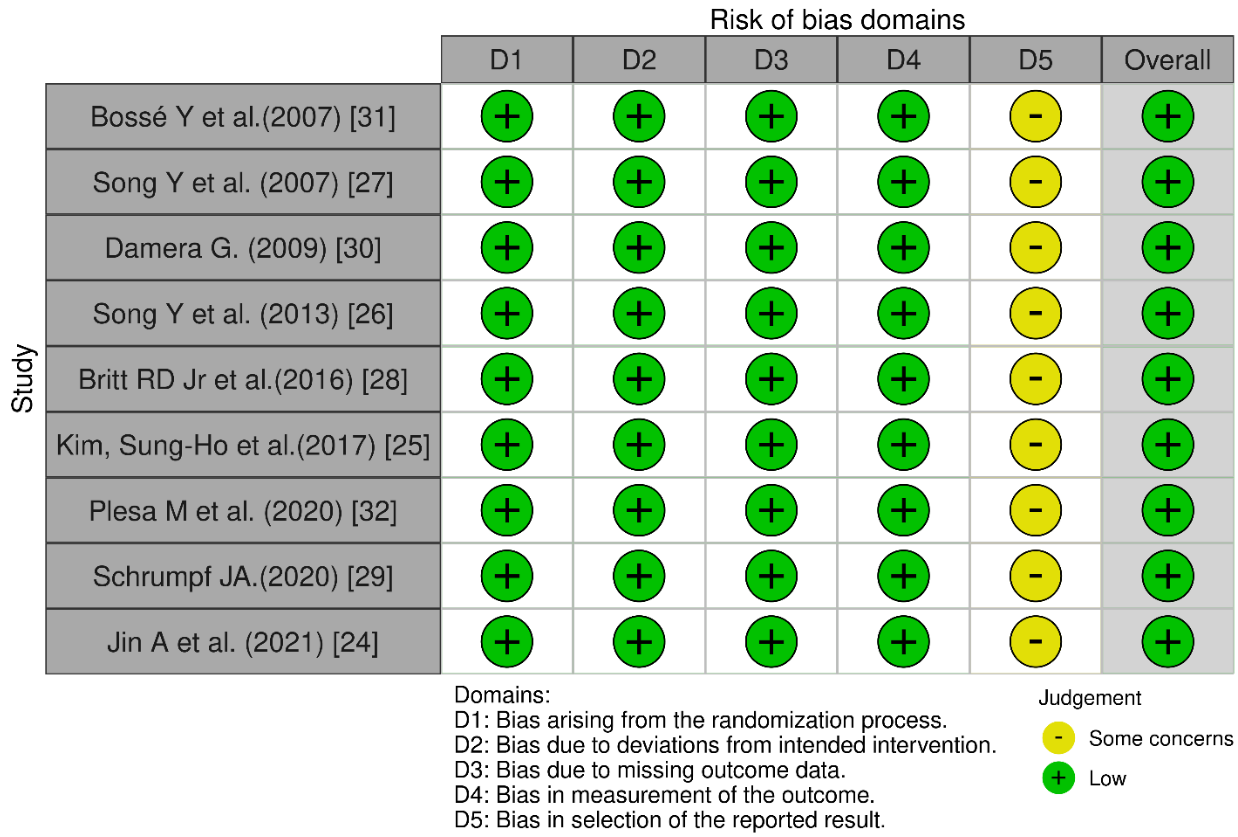

Supplementary Figure S1: Bias assessment of included studies using Revised Cochrane risk-of-bias tool for randomized trials (RoB 2). Assessment of the risk of bias via the Cochrane RoB 2 tool displayed by means of a traffic light plot of the risk of bias of each included clinical study .Traffic light plot reports five risk of bias domains: D1, bias arising from the randomization process; D2, bias due to deviations from intended intervention; D3, bias due to missing outcome data; D4, bias in measurement of the outcome; D5, bias in selection of the reported result; Yellow circle indicates some concerns on the risk of bias and green circle represents low risk of bias. Note: Figure image was created using .

Abbreviation: RoB, risk of bias.
