# Supplementary material for: The Role of Vitamin D Supplementation on Airway Remodeling in Asthma: A Systematic Review
Source: Nutrients. 2023 May 26;15(11):2477. doi: 10.3390/nu15112477 (PMC10255490; doi:10.3390/nu15112477)
Supplement: Supplementary file 1 [file nutrients-15-02477-s001.zip › nutrients-2394072-supplementary table.pdf]

Table S1. Literature search strategy and terms for role of vitamin D on asthma airway remodeling.

Source	The role of vitamin D supplementation on Airway Remodeling in Asthma
<u>**PubMed**</u>	<u>**Key words**</u>
Search #1	((((((((((calcitriol[MeSH Terms]) OR (cholecalciferol[MeSH Terms])) OR (1,25 dihydroxy 20 epi vitamin d3[MeSH Terms])) OR (vitamin D [Title/Abstract]))OR(cholecalciferol[Title/Abstract]))OR (vit d [Title/Abstract])) OR (calcitriol[Title/Abstract]))OR(1,25dihydroxy 20 epi vitamind3[Title/Abstract]))OR(vitamin D[Title/Abstract]))OR (25(OH)D[Title/Abstract])) OR ("25 hydroxy D"[Title/Abstract])
Search #2	((((bronchial asthma[MeSH Terms]) OR (Asthma[Title/Abstract])) OR (wheeze[Title/Abstract])) OR (recurrent wheeze[Mesh Major Topic]))
Search #3	(Airway remodeling[MeSH Terms]) OR airway remodeling[Title/Abstract]))OR (airway smooth muscle[Title/Abstract])) OR (fibrosis[Title/Abstract])) OR (extracellular matrix[Title/Abstract]))
Search #4	(Randomized controlled trial [pt] OR controlled clinical trial [pt] OR randomized [tiab] OR placebo [tiab] OR drug therapy [sh] OR randomly [tiab] OR trial [tiab] OR groups [tiab])
Search #5	Search #1 AND Search #2 AND Search #3 AND Search #4
<u>**Embase**</u>	
Search #1	'Vitamin d'/exp OR 'cholecalciferol derivative'/exp OR 'vitamin deficiency'/exp OR 'vitamin d':ti,ab,kw OR calcitriol:ti,ab,kw OR '25 hydroxyvitamin d':ti,ab,kw OR (25:ti,ab,kw AND oh:ti,ab,kw AND d:ti,ab,kw)
Search #2	

	'asthma'/exp OR 'recurrent wheezing'/exp OR asthma:ti,ab,kw OR 'reactive airway disease':ti,ab,kw OR wheezing:ti,ab,kw
Search #3	'Airway remodeling'/exp OR 'airway remodeling':ti,ab,kw OR ‘airway smooth muscle’ :ti,ab,kw OR ‘collagen deposition’ :ti,ab,kw OR ‘extracellular matrix’ :ti,ab,kw OR ‘fibrosis’:ti,ab,kw
Search #4	'Randomized controlled trial'/exp OR 'randomized controlled trial':ti,ab,kw OR randomization: ti,ab,kw OR placebo:ti,ab,kw
Search #5	Search #1 AND Search #2 AND Search #3 AND Search #4

<u>ClinicalTrials.gov</u>

Search #1	Vitamin D and Asthma
Search #2	Vitamin D and Airway remodeling
Search #3	calcitriol and Asthma
Search #4	calcitriol and Airway remodeling
Search #5	'25 hydroxyvitamin d' and asthma
Search #6	'25 hydroxyvitamin d' and Airway remodeling
Search #7	Cholecalciferol and asthma
Search #8	Cholecalciferol and airway remodeling
Search #9	Vitamin D and wheezing
Search #10	Vitamin D and fibrosis
Search #11	Calcitriol and wheezing
Search #12	Calcitriol and airway remodeling

Search #13	'25 hydroxyvitamin d′ and fibrosis
Search #14	Vitamin D and smooth muscles airway
Search #15	'25 hydroxyvitamin d′ and smooth muscles airway

CINAHL

Search #1	("vitamin D" OR "cholecalciferol" OR "ergocalciferol") AND ("asthma" OR "airway" OR "bronchial") AND ("remodeling" OR "fibrosis") AND ("randomized controlled trial" OR "controlled clinical trial")
**Filter search**	Publication date from January 1, 1990, to February 28, 2023; Adult Humans

Table S2. Standardized checklist for risk bias (Cochrane risk-of-bias tool for randomized trials (RoB 2)).

Revised Cochrane risk-of-bias tool for cluster-randomized trials (RoB 2 CRT)
SHORT VERSION (CRIBSHEET)

Version of 18 March 2021

The development of the RoB 2 tool was supported by the MRC Network of Hubs for Trials Methodology Research (MR/L004933/2- N61), with the support of the host MRC ConDuCT-II Hub (Collaboration and innovation for Difficult and Complex randomised controlled Trials In Invasive procedures - MR/K025643/1), by MRC research grant MR/M025209/1, and by a grant from The Cochrane Collaboration.

This work is licensed under a Creative Commons Attribution-NonCommercial-NoDerivatives 4.0 International License.

Preliminary considerations

Study design

☐ Individually-randomized parallel-group trial
☐ Cluster-randomized parallel-group trial
☐ Individually randomized cross-over (or other matched) trial

For the purposes of this assessment, the interventions being compared are defined as

Experimental: ☐ Comparator: ☐

Specify which outcome is being assessed for risk of bias

Specify the numerical result being assessed. In case of multiple alternative analyses being presented, specify the numeric result (e.g. RR = 1.52 (95% CI 0.83 to 2.77) and/or a reference (e.g. to a table, figure or paragraph) that uniquely defines the result being assessed.

Is the review team's aim for this result…?

☐ to assess the effect of *assignment to intervention* (the 'intention-to-treat' effect)
☐ to assess the effect of *adhering to intervention* (the 'per-protocol' effect)

If the aim is to assess the effect of *adhering to intervention*, select the deviations from intended intervention that should be addressed (at least one must be checked):

☐ occurrence of non-protocol interventions
☐ failures in implementing the intervention that could have affected the outcome
☐ non-adherence to their assigned intervention by trial participants

Which of the following sources were obtained to help inform the risk-of-bias assessment? (tick as many as apply)

☐ Journal article(s)
☐ Trial protocol
☐ Statistical analysis plan (SAP)
☐ Non-commercial trial registry record (e.g. ClinicalTrials.gov record)
☐ Company-owned trial registry record (e.g. GSK Clinical Study Register record)
☐ “Grey literature” (e.g. unpublished thesis)
☐ Conference abstract(s) about the trial
☐ Regulatory document (e.g. Clinical Study Report, Drug Approval Package)
☐ Research ethics application
☐ Grant database summary (e.g. NIH RePORTER or Research Councils UK Gateway to Research)
☐ Personal communication with trialist
☐ Personal communication with the sponsor

Domain 1a: Risk of bias arising from the randomization process

Signalling questions	Elaboration	Response options
**1a.1 Was the allocation sequence random?**	Considerations are mostly the same as for individually randomized trials. Answer 'No' for non-random methods that might be seen in cluster-randomized trials, including those based on geography (e.g. clusters near the main research centre allocated to the intervention and those further away to the control).	Y/PY/PN/N/NI
**1a.2 Was the allocation sequence concealed until clusters were enrolled and assigned to interventions?**	As for individually randomized trials.	Y/PY/PN/N/NI
**1a.3 Did baseline differences between intervention groups suggest a problem with the randomization process?**	*Note that differences that are compatible with chance do not lead to a risk of bias.*<br><br>Answer 'No' if any observed imbalances are compatible with chance or likely to be because of identification/recruitment bias, which are addressed in domain 1b (see section 3).<br><br>Imbalances in numbers of clusters or in stratification/ matching/ minimization factors can provide evidence of problems with the randomization process, but such problems are likely to be unusual in cluster-randomized trials. Due to the small numbers of clusters randomized in most cluster-randomized trials, chance imbalances in either cluster or participant characteristics, which can be substantial, are more common than in individually-randomized trials.<br><br>Answer 'Yes' if there are imbalances that indicate problems with the randomization process, including:<br>(1) substantial differences between numbers of clusters between intervention arms, compared with the intended allocation ratio;<br>or<br>(2) a substantial excess in statistically significant differences in baseline cluster characteristics between intervention groups, beyond that expected by chance; or<br>(3) imbalance in one or more baseline measures of outcome variables, that is very unlikely to be due to chance and for which the between-group difference is big enough to result in bias in the intervention effect estimate.<br><br>Also answer 'Yes' if there are other reasons to suspect that the randomization process was problematic:<br>(4) excessive similarity in baseline characteristics that is not compatible with chance.	Y/PY/PN/N/NI

	Answer 'No information' when there is no *useful* baseline information available (e.g. abstracts, or studies that reported only baseline characteristics of participants in the final analysis).<br><br>In some circumstances, it may be reasonable to answer "Yes/Probably yes" (rather than "No information") when there is a surprising lack of information on baseline characteristics and when such information could reasonably be expected to be available/reported.<br><br>The answer to this question should not be used to influence answers to questions 1a.1 or 1a.2. For example, if the trial has large baseline imbalances that are judged to be unlikely to be due to chance or identification/recruitment bias, but authors report adequate randomization methods, questions 1a.1 and 1a.2 should still be answered on the basis of the reported adequate methods, and any concerns about the imbalance should be raised in the answer to the question 1a.3 and reflected in the domain-level risk of bias judgement).	
**Risk-of-bias judgement**	See algorithm.	Low / High / Some concerns
Optional: What is the predicted direction of bias arising from the randomization process?	If the likely direction of bias can be predicted, it is helpful to state this. The direction might be characterized either as being towards (or away from) the null, or as being in favour of one of the interventions.	NA / Favours experimental / Favours comparator / Towards null /Away from null / Unpredictable

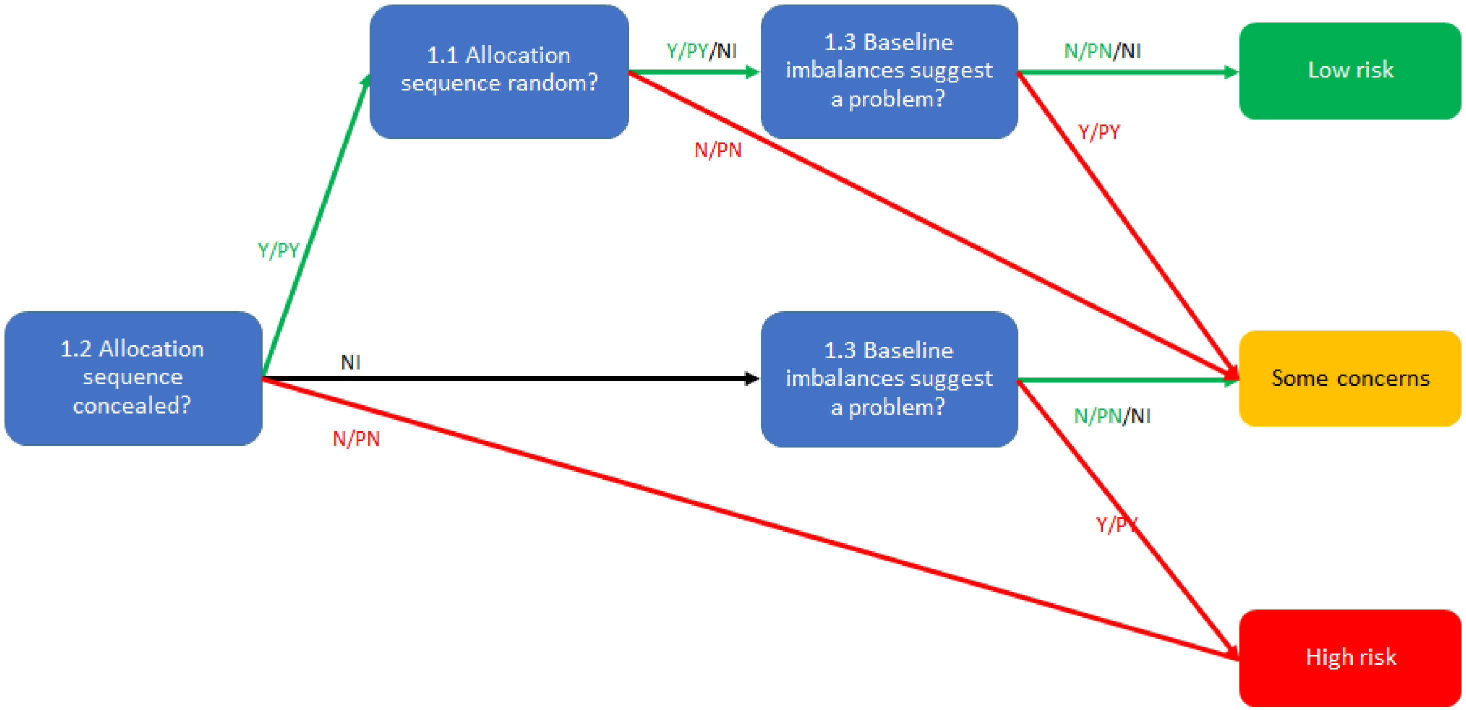

Algorithm for suggested judgement of risk of bias arising from the randomization process

Domain 1b: Risk of bias arising from the timing of identification or recruitment of participants in a cluster-randomized trial

Signalling questions	Elaboration	Response options
**1b.1 Were all the individual participants identified and recruited (if appropriate) before randomization of clusters?**	Answer 'Yes' if:<br>(1) all participants were identified and recruited before the clusters were randomized; or<br>(2) individual participants were not recruited at all but all were identified before randomization.<br>In these cases identification/recruitment bias is not possible.<br><br>Answer 'No' if:<br>(1) some or all participants were identified or recruited after randomization; or<br>(2) there are any clusters in which no participants were recruited (empty clusters).	Y/PY/PN/N/NI
**1b.2 If N/PN/NI to 1b.1: Is it likely that selection of individual participants was affected by knowledge of the intervention assigned to the cluster?**	Answer 'Yes' if:<br><br>(1) those recruiting individuals were aware of cluster allocation before recruitment and this is likely, consciously or subconsciously, to have affected recruitment differentially between the intervention groups;<br><br>(2) some participants were aware of cluster allocation before their recruitment and this is likely to have affected recruitment differentially between the intervention groups; or<br><br>(3) those identifying potential participants (when recruitment is to take place subsequently) are aware of cluster allocation and are likely, consciously or subconsciously, to have differentially included potential individual participants in different trial groups<br><br>or those identifying actual participants (when there is no subsequent recruitment) are aware of cluster allocation and are likely consciously or subconsciously, to have differentially included potential individual participants in different trial groups.<br><br>Answer 'No' if all of the following (as relevant depending on the trial) are unaware of cluster allocation at recruitment:<br>(1) those identifying actual participants,<br>(2) those identifying potential participants;<br>(3) those recruiting; and<br>(4) potential participants.	NA/Y/PY/PN/N/NI

**1b.3 Were there baseline imbalances that suggest differential identification or recruitment of individual participants between intervention groups?**	As for signalling question 1a.3, imbalances that are compatible with chance should not be interpreted as suggesting differential identification or recruitment of participants. Such imbalances are more common in cluster-randomized trials than imbalances due to problems with randomization. They can be in the numbers of participants recruited into each group or in the characteristics of such individuals.	Y/PY/PN/N/NI
**Risk-of-bias judgement**	See algorithm.	Low / High / Some concerns
Optional: What is the predicted direction of bias arising from the timing of identification and recruitment of participants?	If the likely direction of bias can be predicted, it is helpful to state this. The direction might be characterized either as being towards (or away from) the null, or as being in favour of one of the interventions.	NA / Favours experimental / Favours comparator / Towards null /Away from null / Unpredictable

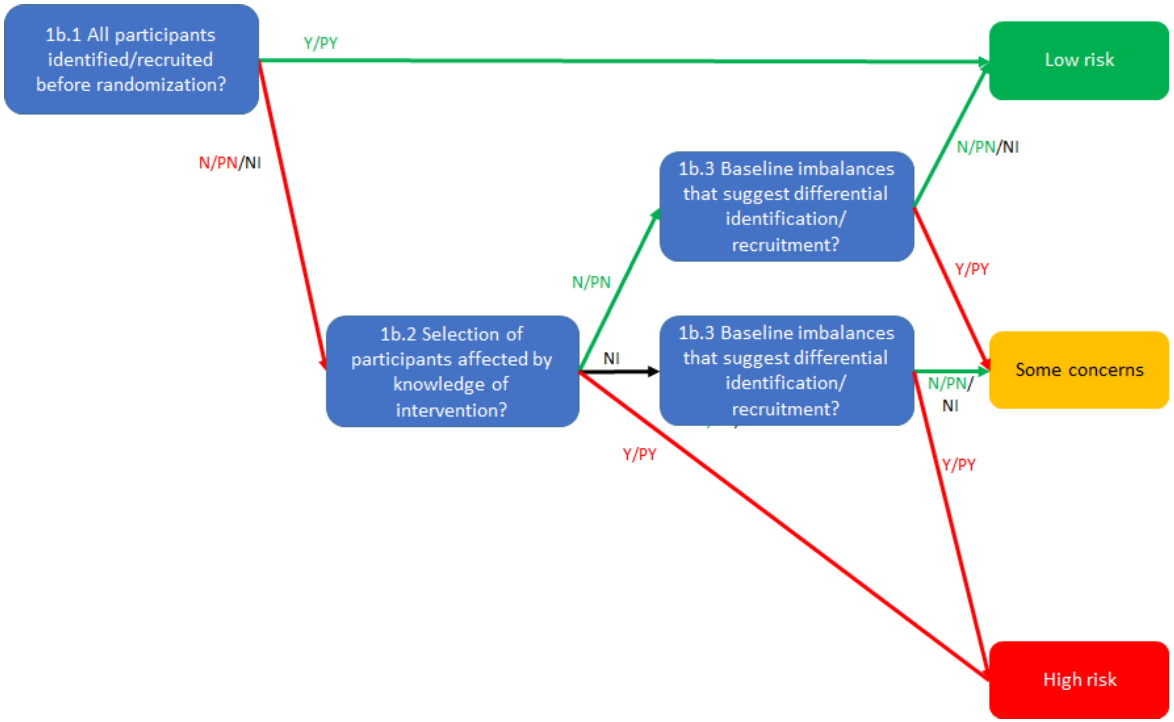

Domain 2: Risk of bias due to deviations from the intended interventions (*effect of assignment to intervention*)

Signalling questions	Elaboration	Response options
**2.1a Were participants aware that they were in a trial?**	In cluster-randomized trials it is possible for participants to know they are receiving an intervention (or even to know that they are in a study) but not to know that they are in a trial. They therefore may not know that another interventions is being compared with theirs or what this other intervention is. This makes it impossible for them to cause deviations from the intended interventions that arise because of the trial context.<br><br>Answer 'No' if participants are not aware that they are in a study or aware that they are in a study but not that they are in trial.	Y/PY/PN/N/NI

**2.1b If Y/PY/NI to 2.1a: Were participants aware of their assigned intervention during the trial?**	Answer 'Yes' if participants were aware of any part of the assigned intervention during the trial. It is important to consider all parts of the assigned intervention. Note that, for the purposes of the risk of bias tool, participants are defined as those on whom investigators seek to measure the outcome under consideration, and may be patients, the public, health professionals or other cluster staff.	NA/Y/PY/PN/N/NI
**2.2. Were carers and people delivering the interventions aware of participants' assigned intervention during the trial?**	If those involved in caring for participants or making decisions about their health care are aware of the assigned intervention, then implementation of the intended intervention, or administration of non-protocol interventions, may differ between the intervention groups. Blinding carers and trial personnel, which is most commonly achieved through use of a placebo, may prevent such differences, but this is rare in cluster randomized trials.	Y/PY/PN/N/NI
**2.3. If Y/PY/NI to 2.1b or 2.2: Were there deviations from the intended intervention that arose because of the trial context?**	The guidance mostly applies as for individually-randomized trials.<br>Deviations from the intended intervention that arise due to the trial context are rarely reported in cluster-randomized trials and may, in fact, occur rarely. This is likely to be partly because in these trials interventions are often aimed at clusters and cluster staff. These staff may not have the authority to introduce deviations, and if they do, may have less motivation to do so than caregivers or participants in individually randomized trials who are more directly aware of the intervention. In addition, the more complex the intervention, the more difficult it might be practically to identify such deviations. The answer 'No information' will therefore be appropriate in many cases, but 'Probably yes' should be used if it seems likely that such deviations occurred.	NA/Y/PY/PN/N/NI
**2.4 If Y/PY to 2.3: Were these deviations likely to have affected the outcome?**	As for individually-randomized trials.	NA/Y/PY/PN/N/NI
**2.5. If Y/PY/NI to 2.4: Were these deviations from intended intervention balanced between groups?**	As for individually-randomized trials.	NA/Y/PY/PN/N/NI
**2.6 Was an appropriate analysis used to estimate the effect of assignment to intervention?**	Answer 'Yes' if all clusters and individuals were analysed according to the groups to which they were assigned. Note that there are various reasons why, in some cluster-randomized trials, it is not possible to identify with certainty the groups to which individuals in the trial were assigned, or whether some individuals change clusters part-way through the trial. If the number of such individuals can reasonably be	Y/PY/PN/N/NI

	expected to be very small and unrelated to the individual's assigned group, an analysis that analyses all individuals in the groups to which they were assigned *as far as possible* should be considered appropriate.<br>When analyses exclude only participants with missing outcome data, these should be considered appropriate with regard to this signalling question: missing outcome data are addressed in a separate domain.<br>Answer 'No' if trial participants were analysed according to the intervention they received, rather than according to the intervention to which they were assigned, or if analyses exclude trial participants or clusters not receiving their assigned intervention., or a stepped wedge trial does not take into account the time trend.<br>Analyses excluding eligible trial participants after randomization should be considered inappropriate, but exclusions of ineligible participants after randomization (when eligibility was not confirmed until after randomization, and could not have been influenced by intervention group assignment) can be considered appropriate.	
**2.7 If N/PN/NI to 2.6: Was there potential for a substantial impact (on the result) of the failure to analyse participants in the group to which they were randomized ?**	As for individually randomized trials but bearing in mind that reviewers need to look out for entire clusters analysed in the wrong intervention group as well as individual participants.	NA/Y/PY/PN/N/NI
**Risk-of-bias judgement**	See algorithm.	Low / High / Some concerns
Optional: What is the predicted direction of bias due to deviations from intended interventions?	If the likely direction of bias can be predicted, it is helpful to state this. The direction might be characterized either as being towards (or away from) the null, or as being in favour of one of the interventions.	NA / Favours experimental / Favours comparator / Towards null /Away from null / Unpredictable

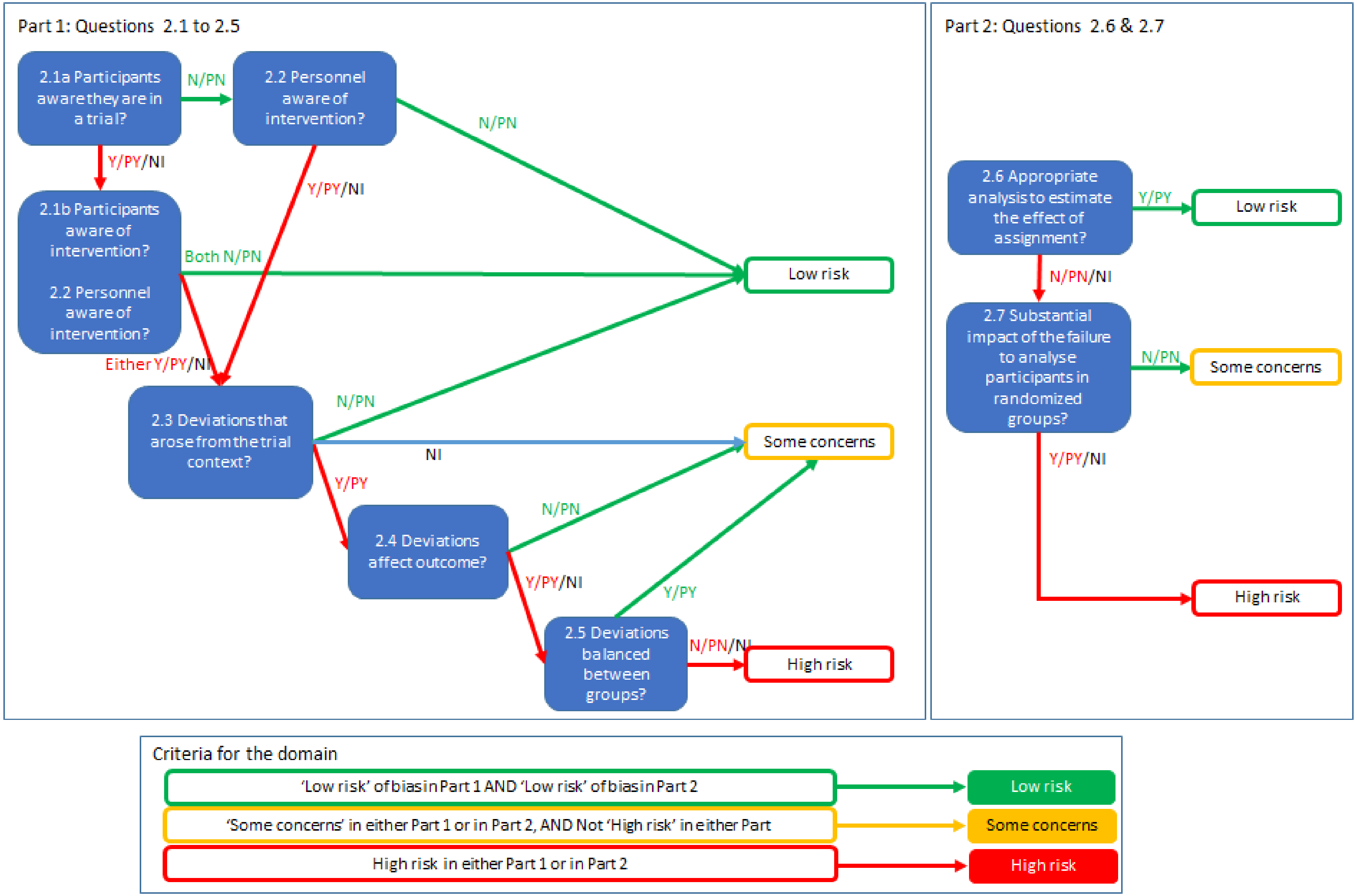

Algorithm for suggested judgement of risk of bias due to deviations from the intended interventions (effect of assignment to intervention)

Domain 2: Risk of bias due to deviations from the intended interventions (*effect of adhering to intervention*)

Signalling questions	Elaboration	Response options
**2.1. Were participants aware of their assigned intervention during the trial?**	If participants are aware of their assigned intervention it is more likely that health-related behaviours will differ between the intervention groups. Blinding participants, most commonly through use of a placebo or sham intervention, may prevent such differences. If participants experienced side effects or toxicities that they knew to be specific to one of the interventions, answer this question 'Yes' or 'Probably yes'.	Y/PY/PN/N/NI
**2.2. Were carers and people delivering the interventions aware of participants' assigned intervention during the trial?**	If carers or people delivering the interventions are aware of the assigned intervention then its implementation, or administration of non-protocol interventions, may differ between the intervention groups. Blinding may prevent such differences. If participants experienced side effects or toxicities that carers or people delivering the interventions knew to be specific to one of the interventions, answer 'Yes' or 'Probably yes'. If randomized allocation was not concealed, then it is likely that carers and people delivering the interventions were aware of participants' assigned intervention during the trial.	Y/PY/PN/N/NI
**2.3. [If applicable:] If Y/PY/NI to 2.1 or 2.2: Were important non-protocol interventions balanced across intervention groups?**	Answer 'Yes' if participants were aware of any part of the assigned intervention during the trial. It is important to consider all parts of the assigned intervention. Note that, for the purposes of the risk of bias tool, participants are defined as those on whom investigators seek to measure the outcome under consideration, and may be patients, the public, health professionals or other cluster staff.	NA/Y/PY/PN/N/NI
**2.4. [If applicable:] Were there failures in implementing the intervention that could have affected the outcome?**	If those involved in caring for participants or making decisions about their health care are aware of the assigned intervention, then implementation of the intended intervention, or administration of non-protocol interventions, may differ between the intervention groups. Blinding carers and trial personnel, which is most commonly achieved through use of a placebo, may prevent such differences, but this is rare in cluster randomized trials.	NA/Y/PY/PN/N/NI
**2.5. [If applicable:] Was there non-adherence to the assigned intervention regimen that could have affected participants' outcomes?**	Mostly as for individually-randomized trials. It is important to consider co-interventions at both the individual and cluster level.	NA/Y/PY/PN/N/NI

**2.6. If N/PN/NI to 2.3, or Y/PY/NI to 2.4 or 2.5: Was an appropriate analysis used to estimate the effect of adhering to the intervention?**	Mostly as for individually-randomized trials. When interventions are multifacteted, it is important to consider all interventions for which implementation failures could have affected the outcome. These include interventions aimed at whole clusters and professionals in clusters, as well as those aimed at individual patients and members of the public.	NA/Y/PY/PN/N/NI
**Risk-of-bias judgement**	See algorithm.	Low / High / Some concerns
Optional: What is the predicted direction of bias due to deviations from intended interventions?	If the likely direction of bias can be predicted, it is helpful to state this. The direction might be characterized either as being towards (or away from) the null, or as being in favour of one of the interventions.	NA / Favours experimental / Favours comparator / Towards null /Away from null / Unpredictable

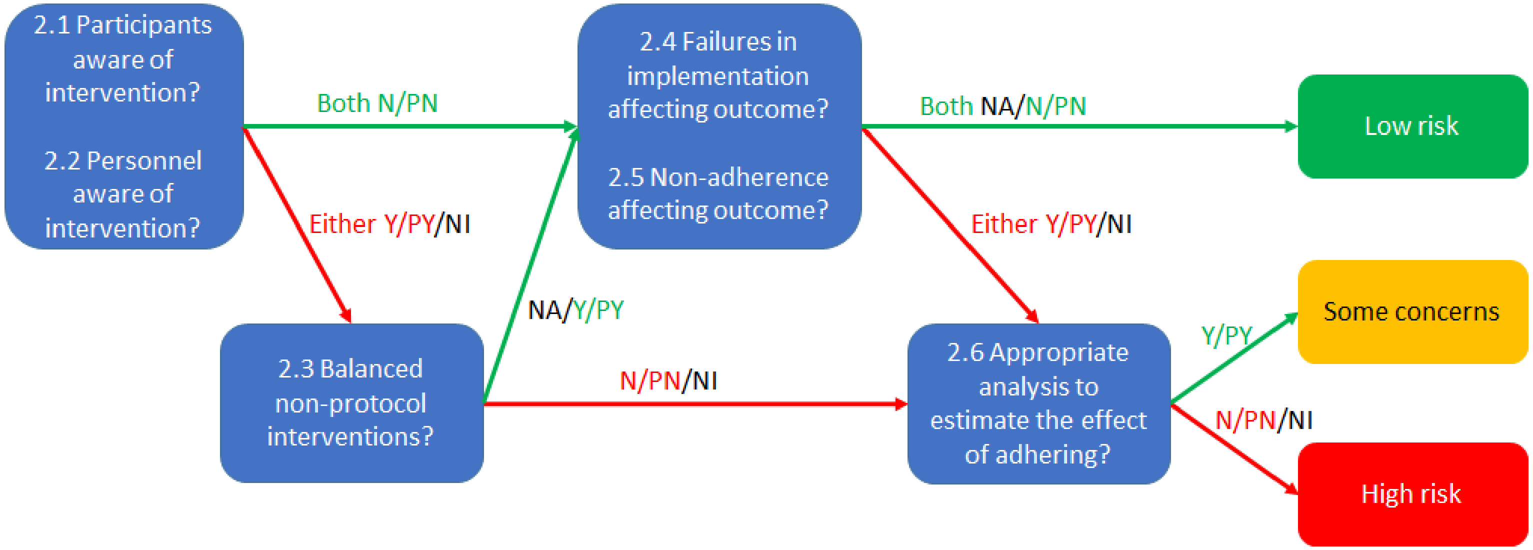

Algorithm for suggested judgement of risk of bias due to deviations from the intended interventions (effect of adhering to intervention)

Domain 3: Risk of bias due to missing outcome data

Signalling questions	Elaboration	Response options
**3.1a Were data for this outcome available for all clusters that recruited participants?**	Note that in some cluster randomized trials there may be some clusters in which no participants are recruited. This can happen only when participants are recruited following randomization and is dealt with in domain 1b. Given that there are usually a relatively small number of clusters in a cluster randomized trial, there is potential for bias in some trials even if only one cluster has no analysable participants.	Y/PY/PN/N/NI
**3.1b Were data for this outcome available for all, or nearly all, participants within clusters?**	The issues here are broadly as for individually-randomized trials. In cluster-randomized trials there may be particular complexities when clusters merge, split, or disappear.	Y/PY/PN/N/NI
**3.2 If N/PN/NI to 3.1a or 3.1b: Is there evidence that the result was not biased by missing data?**	As for individually-randomized trials.	NA/Y/PY/PN/N
**3.3 If N/PN to 3.2 Could missingness in the outcome depend on its true value?**	As for individually-randomized trials.	NA/Y/PY/PN/N/NI
**3.4 If Y/PY/NI to 3.3: Is it likely that missingness in the outcome depended on its true value?**	As for individually-randomized trials.	NA/Y/PY/PN/N/NI
**Risk-of-bias judgement**	See algorithm.	Low / High / Some concerns
Optional: What is the predicted direction of bias due to missing outcome data?	If the likely direction of bias can be predicted, it is helpful to state this. The direction might be characterized either as being towards (or away from) the null, or as being in favour of one of the interventions.	NA / Favours experimental / Favours comparator / Towards null /Away from null / Unpredictable

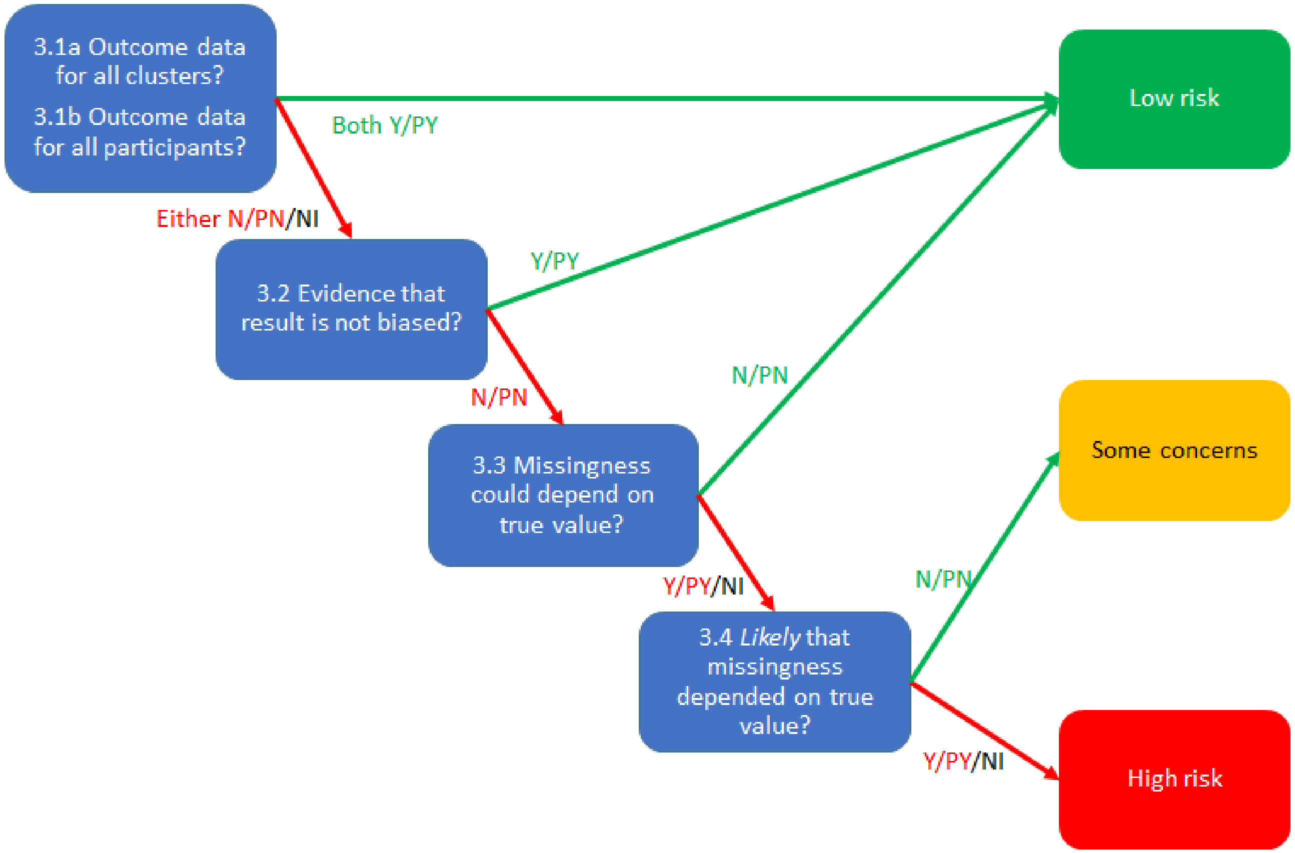

Algorithm for suggested judgement of risk of bias due to missing outcome data

Domain 4: Risk of bias in measurement of the outcome

Signalling questions	Elaboration	Response options
**4.1 Was the method of measuring the outcome inappropriate?**	As for individually randomized trials.	Y/PY/PN/N/NI
**4.2 Could measurement or ascertainment of the outcome have differed between intervention groups?**	As for individually randomized trials.	Y/PY/PN/N/NI
**4.3a If N/PN/NI to 4.1 and 4.2: Were outcome assessors aware that a trial was taking place?**	This question applies to cluster-randomized trials in which participants report their outcomes themselves, for example in a questionnaire. If they are not aware that they are in a trial then their self-assessment cannot be affected by assignment even if they are aware of the intervention they received.	NA/Y/PY/PN/N/NI
**4.3b If Y/PY/NI to 4.3a: Were outcome assessors aware of the intervention received by study participants?**	Answer 'No' if outcome assessors were blinded to intervention status. In studies where participants report their outcomes themselves (i.e., participant-reported outcome), the outcome assessor is the study participant. In cases where outcomes are collected using routine data, the the individual who provides the data (usually patients or clinicians)and the individual responsible for extracting the data can be considered as oucome assessors.	NA/Y/PY/PN/N/NI
**4.4 If Y/PY/NI to 4.3b: Could assessment of the outcome have been influenced by knowledge of intervention received?**	As for individually-randomized trials.	NA/Y/PY/PN/N/NI
**4.5 If Y/PY/NI to 4.4: Is it likely that assessment of the outcome was influenced by knowledge of intervention received?**	As for individually-randomized trials.	NA/Y/PY/PN/N/NI

**Risk-of-bias judgement**	See algorithm.	Low / High / Some concerns
Optional: What is the predicted direction of bias in measurement of the outcome?	If the likely direction of bias can be predicted, it is helpful to state this. The direction might be characterized either as being towards (or away from) the null, or as being in favour of one of the interventions.	NA / Favours experimental / Favours comparator / Towards null /Away from null / Unpredictable

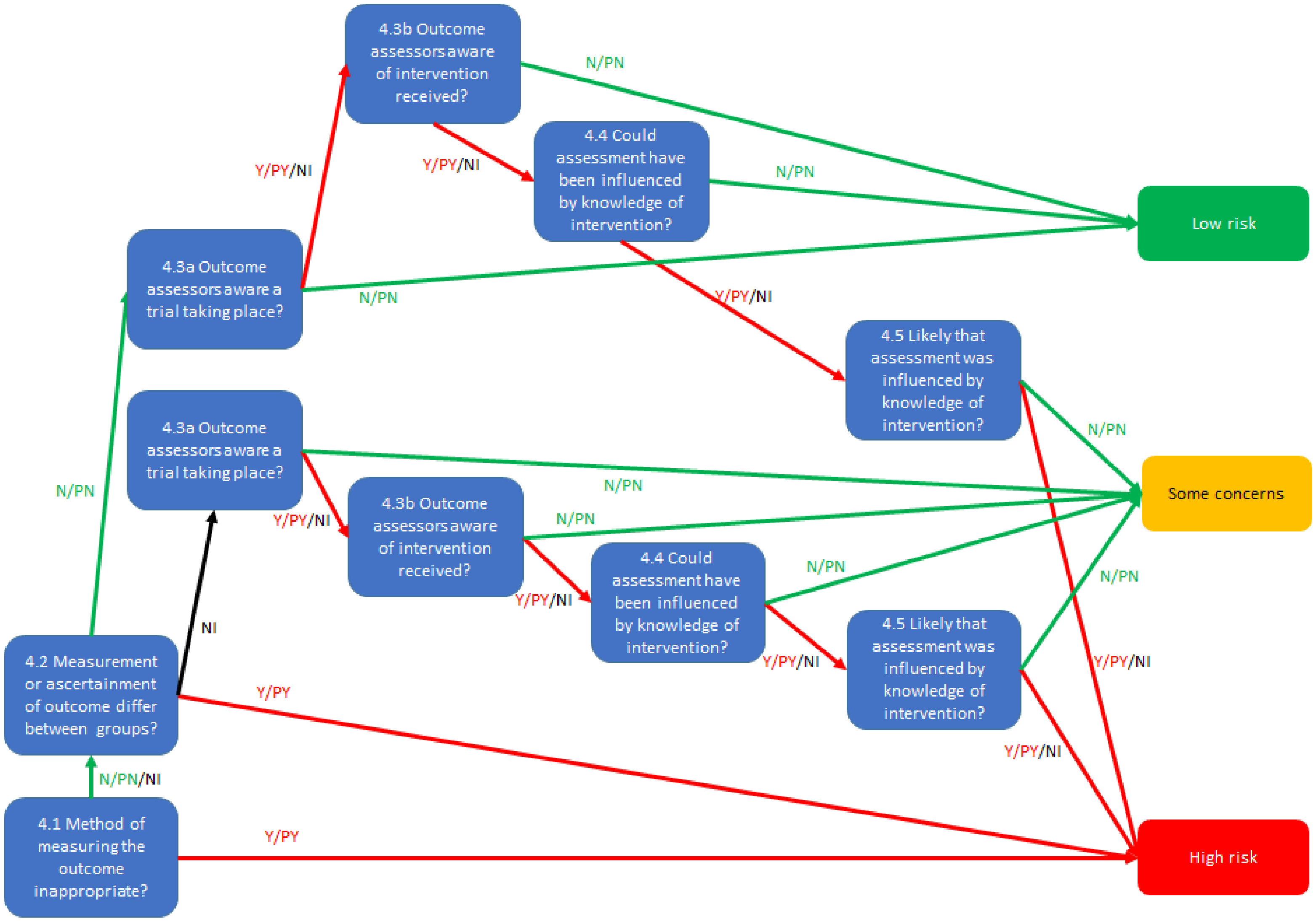

Algorithm for suggested judgement of risk of bias in measurement of the outcome

Domain 5: Risk of bias in selection of the reported result

Signalling questions	Elaboration	Response options
**5.1 Were the data that produced this result analysed in accordance with a pre-specified analysis plan that was finalized before unblinded outcome data were available for analysis?**	As for individually-randomized trials.	Y/PY/PN/N/NI
**Is the numerical result being assessed likely to have been selected, on the basis of the results, from...**		
**5.2. ... multiple eligible outcome measurements (e.g. scales, definitions, time points) within the outcome domain?**	As for individually-randomized trials.	Y/PY/PN/N/NI
**5.3 ... multiple eligible analyses of the data?**	As for individually-randomized trials.	Y/PY/PN/N/NI
**Risk-of-bias judgement**	See algorithm.	Low / High / Some concerns
Optional: What is the predicted direction of bias due to selection of the reported result?	If the likely direction of bias can be predicted, it is helpful to state this. The direction might be characterized either as being towards (or away from) the null, or as being in favour of one of the interventions.	NA / Favours experimental / Favours comparator / Towards null /Away from null / Unpredictable

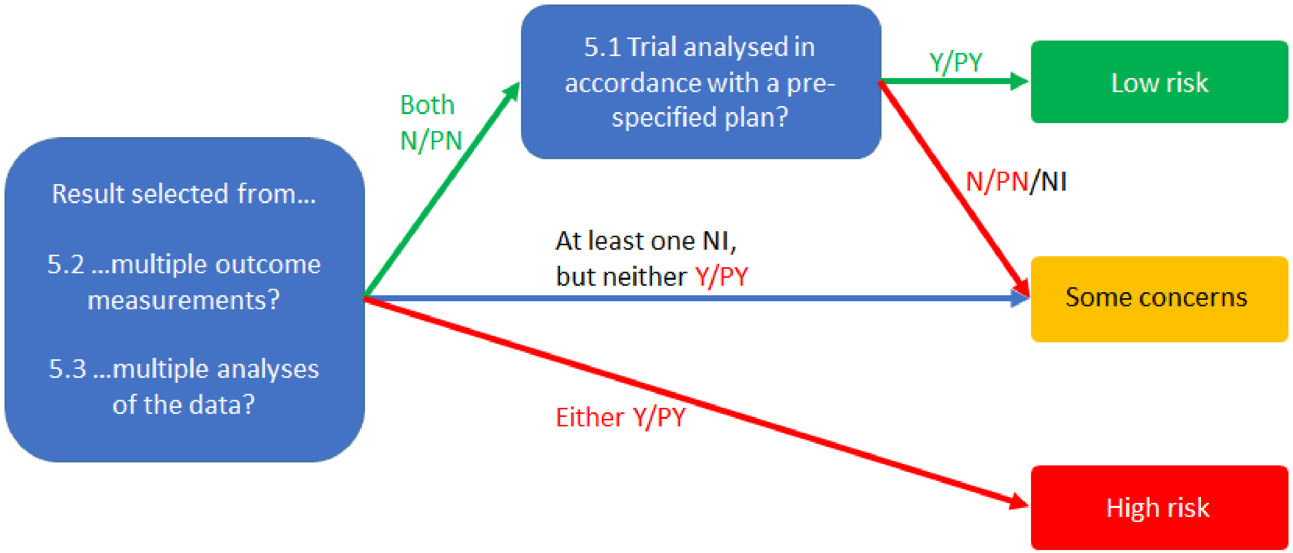

Algorithm for suggested judgement of risk of bias in selection of the reported result

Overall risk of bias

**Risk-of-bias judgement**		Low / High / Some concerns
Optional: What is the overall predicted direction of bias for this outcome?		Favours experimental / Favours comparator / Towards null /Away from null / Unpredictable / NA

**Overall risk-of-bias judgement**	**Criteria**
Low risk of bias	The study is judged to be at **low risk of bias for all domains** for this result.
Some concerns	The study is judged to raise **some concerns** in at least one domain for this result, but not to be at high risk of bias for any domain.
High risk of bias	The study is judged to be at **high risk of bias** in at least one domain for this result.<br>Or<br>The study is judged to have **some concerns** for **multiple domains** in a way that substantially lowers confidence in the result.

The development of the RoB 2 tool was supported by the MRC Network of Hubs for Trials Methodology Research (MR/L004933/2- N61), with the support of the host MRC ConDuCT-II Hub (Collaboration and innovation for Difficult and Complex randomised controlled Trials In Invasive procedures - MR/K025643/1), by MRC research grant MR/M025209/1, and by a grant from The Cochrane Collaboration.

This work is licensed under a Creative Commons Attribution-NonCommercial-NoDerivatives 4.0 International License.
